# Genomic Epidemiology and Clinical Characteristics of Mpox Lineage C.1 Outbreak in Thailand, 2023–2024

**DOI:** 10.1002/jmv.71142

**Published:** 2026-09-08

**Authors:** Kamol Suwannakarn, Chutikarn Chaimayo, Atsadang Boonmee, Charussri Leeyaphan, Pattriya Jirawattanadon, Pravarit Athiprayoon, Natsada Saengdao, Piyarat Taiphakphaibun, Chutima Deetippasert, Warinya Nuwong, Niracha Athipanyasilp, Archiraya Pattama, Navin Horthongkham

**Affiliations:** ^1^ Department of Microbiology, Faculty of Medicine Siriraj Hospital Mahidol University Bangkok Thailand; ^2^ Department of Dermatology, Faculty of Medicine Siriraj Hospital Mahidol University Bangkok Thailand

**Keywords:** genomic epidemiology, human monkeypox virus, lineage C.1, local transmission, Mpox, whole‐genome sequencing

## Abstract

Since 2022, human monkeypox virus (hMPXV) has emerged in non‐endemic regions, including Thailand. However, the genomic dynamics and clinical correlates of local transmission remain incompletely defined. Whole‐genome sequencing was performed on hMPXV from 16 patients in Thailand (2023–2024) using targeted amplicon NGS. Phylogenetic analyses integrated global reference sequences. Mutational profiles, specifically non‐synonymous substitutions and APOBEC3‐associated signatures, were analyzed in relation to clinical data. Phylogenetic reconstruction identified three temporal phases. Early 2022 cases (clade IIb lineages A and B) were interspersed with global sequences, consistent with multiple introductions. In contrast, 2023–2024 cases were dominated by lineage C.1. All 16 genomes belonged to C.1 (one C.1.1), and formed a distinct mid‐2023 cluster, designated C.1/Thai/Cluster, supporting sustained local transmission. APOBEC3‐associated mutations were pervasive across the C.1 lineage overall, including within C.1/Thai/Cluster, without evidence of significant enrichment specific to this cluster. The cohort comprised exclusively male patients (81% HIV‐positive, MSM), with predominantly genital painful lesions and a median recovery time of 23 days. No significant associations were detected between viral genetic variation and clinical outcomes. Mpox transmission in Thailand evolved from multiple introductions to sustained C.1‐dominated local spread, underscoring the importance of continued genomic surveillance.

## 1 Introduction

Mpox is a zoonotic disease caused by the human monkeypox virus (hMPXV), a member of the family *Poxviridae* and the genus *Orthopoxvirus*. This genus comprises 13 recognized species, among which hMPXV is one of the most clinically significant [1]. The virus is enveloped and possesses a linear double‐stranded DNA genome of approximately 197 kilobases. The hMPXV was identified in 1958 during outbreaks of a pox‐like disease among colonies of captive monkeys maintained for research in Copenhagen, Denmark [2], and the first human infection was reported in the Democratic Republic of Congo in 1970 [3]. Since then, the virus has been detected across Central and Southern Africa and has subsequently spread to regions outside the African continent [4]. Human infections are primarily transmitted through direct contact with infected individuals, zoonosis through contact with particular rodents, or contaminated materials. In May 2022, a large multicounty outbreak of mpox emerged, leading the World Health Organization (WHO) to declare it a Public Health Emergency of International Concern (PHEIC) in July 2022 [5]. This outbreak differed from previous ones, as transmission occurred predominantly through sexual contact, especially among men who have sex with men (MSM) [6]. From 2022 to 2024, more than 115,101 confirmed mpox cases were reported worldwide [7].

The hMPXV are classified into two major clades based on geographic and genetic distinctions: Clade I (Congo Basin clade) and Clade II (West African clade). Clade I is further divided into subclades Ia and Ib, whereas Clade II comprises IIa and IIb, the latter being responsible for the 2022 global outbreak [8]. Recent genomic analyses revealed that viruses with Clade IIb exhibit a high frequency of GA to AA and TC to TT transitions, consistent with APOBEC3 cytidine deaminase activity [9]. These mutational signatures were prominently observed in clade IIb viruses associated with sustained human transmission and have subsequently also been documented in human transmission chains involving clades Ia and Ib [10]. Their progressive accumulation may increase the apparent rate of viral evolution and provide genomic evidence of sustained human‐to‐human transmission. However, data on region‐specific viral evolution and its potential clinical relevance remain limited, particularly concerning the viral strains responsible for outbreaks in Southeast Asia.

Thailand serves as a major international travel hub in Southeast Asia and received approximately 11 million tourists in 2022 and 2023 after reopening post‐COVID‐19 [11]. The first mpox case in Thailand in 2022 involved a traveler from Nigeria [12], followed by a second case in a Thai national with a history of sexual contact with a foreign partner. According to the Department of Disease Control, the circulating strains in 2022 and 2023 belonged to Clade II.

Although numerous hMPXV genome sequences from Thailand have been deposited in public international databases such as GISAID, comprehensive analyses integrating genome characteristics with patient‐level clinical data remain scarce. Understanding the relationship between viral genetic variations and clinical outcomes is essential for elucidating the pathogenesis and transmission dynamics of mpox. Specifically, whether region‐specific mutations circulating in this setting influence disease severity of distinct clinical presentations is currently unknown. Therefore, this study aims to perform whole‐genome sequencing of hMPXV isolated from patients diagnosed at Siriraj Hospital, Bangkok, Thailand, between 2023 and 2024, and to explore potential, hypothesis‐generating associations between genomic mutations and clinical manifestations. By addressing this gap, this information provides updated genomic surveillance data and clinical insights crucial for monitoring viral evolution and optimizing public health strategies in major Southeast Asian hub.

## 2 Materials and Methods

### 2.1 Samples

A total of 16 skin lesion specimens collected in viral transport medium (VTM) from laboratory‐confirmed mpox patients were included in this study. The study covered the period from January 1, 2023, to December 31, 2024, at the Department of Microbiology, Faculty of Medicine Siriraj Hospital. During this period, specimens from 47 patients with suspected mpox were submitted for diagnostic testing using a real‐time PCR assay targeting the hMPXV genome. The hMPXV was detected in 23 patients, including 18 of 24 suspected cases tested in 2023 and 5 of 23 tested in 2024. Among the 23 laboratory‐confirmed cases, specimens with a cycle threshold (Ct) value of < 30 and sufficient residual specimen volume were eligible for whole‐genome sequencing. Sixteen specimens met these criteria and were included in the study, representing 69.6% of all laboratory‐confirmed mpox cases identified at the hospital during the study period. The study protocol was approved by the Siriraj Institutional Review Board, Mahidol University (COA 941/2024). The requirement for informed consent was waived because all specimens and associated clinical data were anonymized, and all procedures were conducted in accordance with relevant institutional guidelines and the principles of the Declaration of Helsinki.

### 2.2 Total Nucleic Acid Extraction

Total nucleic acids were extracted by using the MagLEAD 12gC automated extraction platform (Precision System Science, Chiba, Japan). Briefly, 200 µL of the swab specimen was mixed with 200 µL of lysis buffer and processed according to the manufacturer's protocol for the final elution volume of 100 µL. The extracted nucleic acid was subsequently used for genome copy number quantification using digital droplet PCR (ddPCR) and for whole genome sequencing by next‐generation sequencing.

### 2.3 Real‐Time PCR for hMPXV Detection

Diagnostic confirmation of mpox was performed using the Monkeypox Virus Real Time PCR Kit (Jiangsu Bioperfectus Technologies Co. Ltd., Taizhou, China), according to the manufacturer's instructions. The assay targets the MPXV F3L gene and includes human RNase P as an internal control. Amplification was performed using the CFX96 Real‐Time PCR Detection System (Bio‐Rad Laboratories, Hercules, CA, USA) under the manufacturer‐recommended cycling conditions. A specimen was considered positive when the MPXV target had a cycle threshold (Ct) value of ≤ 40 and all assay controls were valid.

### 2.4 Genomic Copy Number Quantification by Droplet Digital PCR (ddPCR)

Quantification of hMPXV genome copies was performed using the QX200 Droplet Digital PCR System (Bio‐Rad Laboratories, Hercules, CA, USA). in accordance with the manufacturer's instructions. Each 20 µL reaction mixture contained ddPCR Supermix for Probes (no dUTP), optimized concentrations of Mpox 2 G primer/probe mix, and nuclease‐free water. 5 µL of DNA template was added. The prepared master mixes were loaded into a droplet generation plate, and droplets were generated using droplet generation oil for probes and the QX200 Droplet Generator (Bio‐Rad Laboratories). The resulting droplets were transferred to a clean 96‐well ddPCR plate, sealed, and subjected to thermal cycling using a T100™ Thermal Cycler (Bio‐Rad Laboratories) with the following conditions: initial enzyme activation at 95°C for 10 min; 40 cycles of denaturation at 94°C for 30 s and annealing/extension at 60°C for 1 min; followed by final enzyme deactivation at 98°C for 10 min; and a final hold at 4°C.

After amplification, droplet fluorescence was measured using the QX200 Droplet Reader (Bio‐Rad Laboratories), and data were analyzed with QX Manager Software version 2.2 (Bio‐Rad Laboratories) to quantify the number of target nucleic acid copies per microliter (copies/µL) in each well. Thresholds were manually set for each experiment based on the fluorescence intensities of the negative control and the positive control. Droplets with fluorescence values above the set threshold were classified as positive.

### 2.5 Genome Sequencing by Next Generation Sequencing

Whole‐genome sequencing of hMPXV was performed using the ATOPlex MPXV panel (MGI, Shenzhen, China) according to the manufacturer's instructions. Briefly, 10 μL of extracted DNA was subjected to the ATOPlex Multiplex PCR Amplification Kit (MGI, Shenzhen, China), which includes 739 pairs of PCR primers designed to amplify short DNA fragments ranging from 313 to 395 base pairs (bp). The amplification process was carried out using PCR cycles. The resulting PCR products were purified using the MGIEasy DNA Clean Beads (MGI, Shenzhen, China) and quantified using a Qubit fluorometer (Thermo Fisher Scientific, USA). For library preparation, the MGIEasy Fast PCR‐Free FS DNA Library Prep Kit (MGI, Shenzhen, China) was used, involving steps such as enzymatic fragmentation, end repair, adapter ligation, and library purification. Subsequently, the libraries were circularized using the DNBSEQ OneStep DNB Make Reagent Kit (MGI, Shenzhen, China) to generate DNA nanoballs (DNBs). Sequencing was carried out on the DNBSEQ‐G99 platform using paired‐end 150‐bp reads (PE150), following the manufacturer's standard protocol.

Raw reads were assessed for quality using FastQC [13]. High‐quality reads with Phred scores > 30 were mapped to the hMPXV reference genome (GenBank accession NC_063383.1) using Bowtie2 v2.5.4 [14]. SAM files were converted to BAM format, sorted, and indexed using SAMtools v1.2.1 [15]. Variants were identified using BCFtools v1.2.1 [16], and consensus sequences were generated using SAMtools mpileup, with nucleotides called at positions with a sequencing depth greater than 30× and a depth proportion greater than 50%. Using these criteria, all 16 consensus genomes achieved a horizontal coverage exceeding 99% relative to the reference genome. Clade and lineage assignments were determined using NextClade [17].

### 2.6 Phylogenetic and Mutation Analysis

Consensus genomes obtained in this study were aligned with representative global hMPXV genome retrieved from GenBank and GISAID. Multiple sequence alignment was performed using MAFFT v7.526 with FFT‐NS‐2 algorithm [18] The maximum likelihood phylogenetic tree was constructed with IQ‐TREE 2 v2.40 [19] using best‐fit nucleotide substitution model identified by ModelFinder and node support estimated with 1,000 ultrafast bootstrap replicates. Tree visualization and annotation were carried out using Interactive Tree of Life (iTOL) [20].

To infer the temporal phylogenetic structure of hMPXV lineage C.1 circulating in Thailand, a time‐resolved phylogenetic analysis was performed using TreeTime [21]. Thai hMPXV genomes from this study were analyzed together with publicly available Thai and global C.1 reference genomes. Sequences were aligned to a reference hMPXV genome using standard multiple sequence alignment, followed by masking of low‐quality and ambiguous sites. An initial maximum‐likelihood phylogeny was constructed and used as input for TreeTime, together with sampling dates based on specimen collection times. TreeTime was applied under a strict molecular clock framework to estimate time‐scaled branch lengths and divergence times, with automatic rooting and clock optimization. The resulting dated phylogeny was visualized and interpreted to assess the temporal clustering of Thai C.1 genomes in the context of regional and global viral diversity.

Single‐nucleotide polymorphisms (SNPs) were identified from variant call files (VCFs) and functionally annotated using SnpEff v5.2 [22] to predict the impact of each mutation on coding sequences and proteins. Mutation profiling included detection of synonymous and nonsynonymous substitutions as well as analysis of APOBEC3‐associated mutational signatures (GA → AA and TC → TT). Identified mutations were compared with lineage‐defining markers previously reported for hMPXV clade IIb to determine genetic relationships and evolutionary characteristics.

### 2.7 Statistical Analysis

Associations between viral mutation groups and clinical outcomes were assessed using statistical methods appropriate for small sample sizes. Mutation groups were defined as the presence of at least one selected non‐synonymous mutation within predefined categories. For binary outcomes, associations with mutation groups were evaluated using Fisher's exact test (two‐sided), and effect sizes were reported as odds ratios (ORs). To account for zero‐cell counts in sparse contingency tables, the Haldane–Anscombe correction was applied.

Adjustment for multiple comparisons was performed using the Benjamini–Hochberg false discovery rate (FDR) procedure, with adjusted p‐values reported as q values. Comparisons involving continuous variables, including mutation burden, PCR cycle threshold (Ct) values, and absolute viral load estimated by ddPCR (log10 copies/mL), were conducted using the Mann–Whitney *U* test (two‐sided). All analyses were considered exploratory given the limited sample size.

## 3 Results

During the study period, 23 mpox cases were laboratory confirmed at Siriraj Hospital, of which 16 were included in whole‐genome sequencing, representing 69.6% of the hospital‐confirmed cases. National surveillance data recorded 676 confirmed mpox cases in Thailand in 2023 and 176 cases in 2024, for a total of 852 cases during the same period. Therefore, the 16 sequenced cases represented approximately 1.9% of all nationally reported mpox cases in Thailand during 2023–2024 [23].

### 3.1 Clinical and Demographic Characteristics of the Study Population

The clinical and demographic characteristics of the 16 patients included in the genomic analysis are summarized in Table 1. All patients were male, with a mean age of 39.29 years. Fifteen patients identified as homosexual (15/16, 93.75%), and one identified as bisexual (1/16, 6.25%). Thirteen patients were living with HIV (13/16, 81.25%), including three who were newly diagnosed. One HIV‐negative patient was receiving HIV pre‐exposure prophylaxis. Among the 13 patients with HIV, 10 had suppressed HIV RNA levels of < 40 copies/mL, whereas three had detectable viremia. CD4 + T‐cell counts within 1 year before mpox diagnosis were available for 10 patients, of whom five had counts < 500 cells/mm^3^. Concurrent infections included syphilis in 9 patients (56.25%), hepatitis B virus infection in 4 (25.00%), hepatitis C virus infection in 3 (18.75%), and tuberculosis in 1 (6.25%).

**Table 1:** Clinical and demographic characteristics of patients with Mpox included in the genomic analysis.

Characteristics	Amount
Number of patients	16
Age	
Mean ± SD	39.29 ± 8.94
Median (IQR)	35.75 (32.68–46.80)
Gender	
Male	16 (100%)
Self‐reported sexual orientation	
Homosexual	15 (93.75%)
Bisexual	1 (6.25%)
Comorbidity	
HIV	13 (81.25%)
HBV	4 (25.00%)
HCV	3 (18.80%)
Tuberculosis	1 (6.25%)
Syphilis	9 (56.25)
Clinical sign and symptoms	
Pain at lesion	12 (75.00%)
Fever	8 (50.00%)
Myalgia	4 (25.00%)
Lymphadenopathy	7 (43.75%)
Fatigue	2 (12.5%)
Headache	1 (6.25%)
Sore throat	1 (6.25%)
Genital discharge	1 (6.25%)
Oral ulcer	0 (0%)
Skin lesions	
Umbilicated pustules	8 (50.00%)
Ulcers	7 (43.50%)
Pustules	4 (25.00%)
Papules	3 (18.75%)
Plaque	2 (12.5%)
Vesicle	1 (6.25%)
Erosion	1 (6.25%)
Distribution of lesions	
Genitalia	11 (68.75%)
Trunk	9 (56.25%)
Face	6 (37.5%)
Back	6 (37.5%)
Arm	6 (37.5%)
Perineal area	6 (37.5%)
Legs	5 (31.25%)
Buttock	4 (25.00%)
Neck	3 (18.75%)
Hands	3 (18.75%)
Mons pubis	2 (12.50%)
Scalp	1 (6.25%)
Mouth	1 (6.25%)
Axilla	1 (6.25%)
Foot	1 (6.25%)
Laboratory result	
HIV viral load < 40 copies/mL	10/13 (81.25%)
CD4 level > 500 cells/mm^3^ (within 1 year)	5/10 (50.00%)
MPXV viral load > 10^6^ copies/mL	15/16 (93.75%)
Level of care	
Outpatients	12 (75.00%)
Hospitalization	4 (25.00%)
Antiviral treatment	
Tecovirimat	4 (25%)
Duration of tecovirimat (Mean ± SD)	23.0 ± 13.7
Valaciclovir	1 (6.25%)
Acyclovir	1 (6.25%)
Outcome of Mpox infection	
Improved	15 (93.75%)
Lost to follow‐up	1 (6.25%)

The most frequent clinical manifestations were umbilicated pustules and fever, each occurring in eight patients (50.00%), followed by ulcers and lymphadenopathy, each occurring in seven patients (43.75%). Papules, plaques, and vesicles were observed in three (18.75%), two (12.50%), and one patient (6.25%), respectively. Lesions most commonly involved the genital area in 11 patients (68.75%) and the trunk in nine (56.25%). Facial, back, arm, and perianal/perineal lesions were each observed in six patients (37.50%). Painful lesions were reported in 12 patients (75.00%) (Table 1).

Absolute viral genome quantification by ddPCR demonstrated high viral DNA levels in the analyzed specimens. Fifteen of the 16 samples (93.8%) had viral loads exceeding 10^6^ copies/mL. The median viral load was 1.45 × 10^8^ copies/mL (IQR, 3.89 × 10^7^–3.17 × 10^8^), with a geometric mean of 9.36 × 10^7^ copies/mL. The characteristics of each specimen (Ct value, hMPXV viral load) are presented in Supporting Data 1.

Most cases were managed on an outpatient basis (12/16, 75%), with four patients requiring hospitalization. Tecovirimat therapy was administered to all hospitalized patients, with no significant difference in time to clinical resolution compared to untreated individuals. The overall median time to recovery was 23 days (Supporting Figure 1). Acyclovir or valacyclovir was prescribed to patients with suspected herpes simplex virus co‐infection.

### 3.2 Phylogenetic Analysis of hMPXV in Thailand: An Overview

**Figure 1:**
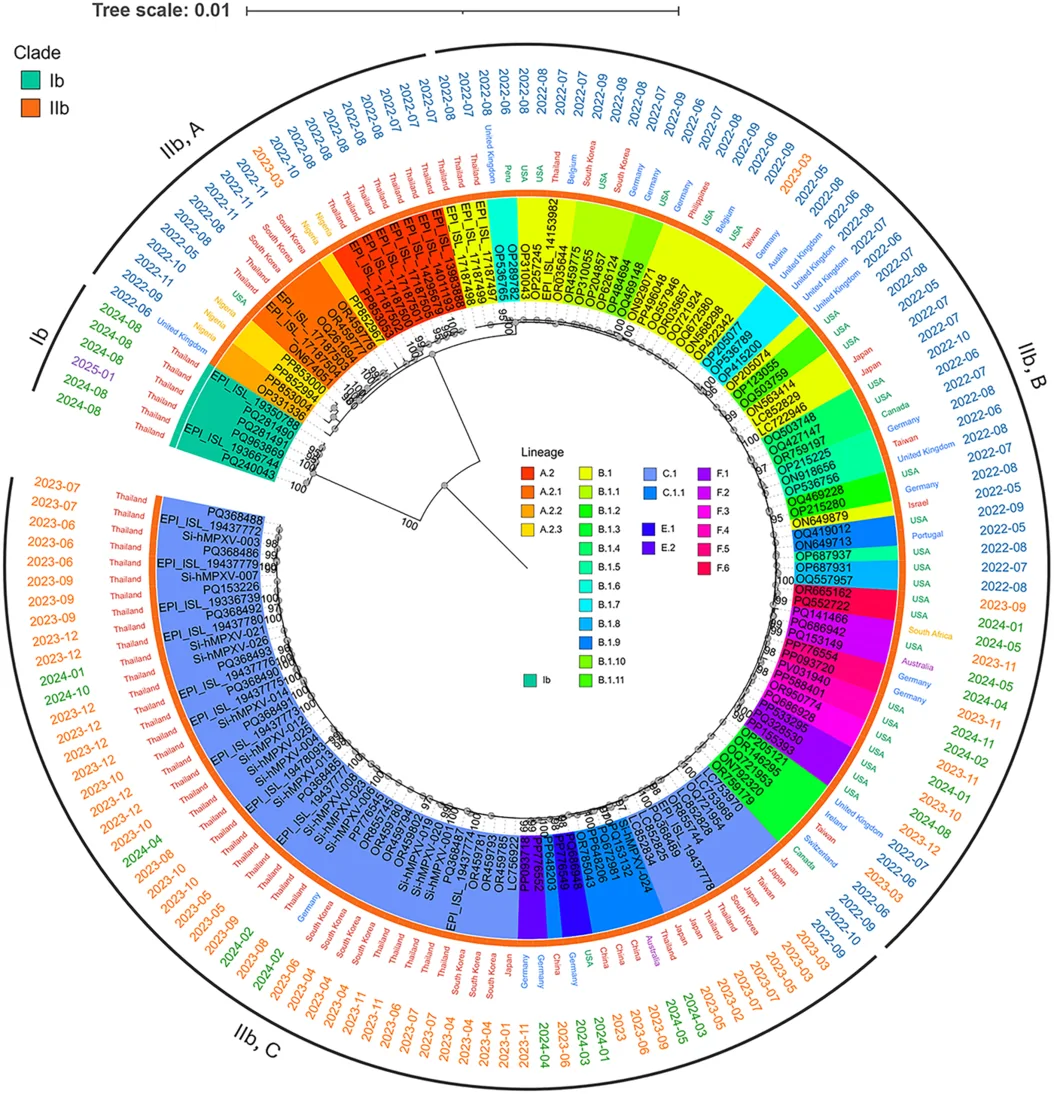
Phylogenetic overview of hMPXV detected in Thailand. Maximum‐likelihood phylogenetic tree based on whole‐genome sequences of hMPXV clade IIb, including Thai isolates from this study (Si‐hMPXV) (*n* = 16), publicly available Thai hMPXV genomes from 2022 to 2024–2025 (*n* = 40), and representative global reference genomes (*n* = 96) from GISAID and GenBank. Branch lengths are proportional to nucleotide substitutions per site (scale bar = 0.01). Tip labels indicate sampling date and country. Bootstrap support values (≥ 95 only) are shown at internal nodes. Colored outer rings denote lineage assignments (A, B, C, E, F, and Ib and sublineages).

To investigate the genomic epidemiology and molecular evolutionary relationships of hMPXV circulating in Thailand, complete whole‐genome sequences were successfully recovered from 16 clinical specimens collected during 2023 to 2024. To contextualize these sequences within Thailand's broader hMPXV epidemiological timeline, we analyzed them together with publicly available Thai hMPXV genomes from 2022 to 2025 (*n* = 40), as well as global reference sequences (*n* = 96) retrieved from GenBank and GISAID (Supporting Data 2). Maximum‐likelihood phylogenetic trees were reconstructed based on whole‐genome alignments to resolve evolutionary relationships and temporal clustering among Thai isolates in a global context (Figure 1).

The comparative phylogenetic analysis, based on publicly available Thai hMPXV sequences deposited in GISAID/GenBank, revealed that hMPXV transmission in Thailand occurred across three temporally and genetically distinct phases. The first phase, corresponding to the early introduction period between July and August 2022, comprised clade IIb lineages A and B, including sub‐lineages A.2, A.2.1, and B.1. Thai genomes from this phase were interspersed among global reference sequences, without forming a distinct phylogenetic cluster, consistent with multiple independent importation events and limited onward local transmission (Figure 1). The second phase began in May 2023 and extended through 2024, representing the major local outbreak period in Thailand. All Thai hMPXV genomes from this phase were classified within clade IIb lineage C.1 and its descendant sub‐lineage C.1.1. Notably, all 16 hMPXV genomes generated in this study clustered within this second phase and were assigned to lineage C.1 and C.1.1. These Thai C.1 genomes clustered with contemporaneous sequences from multiple Asian countries, reflecting regional circulation of C.1 during the 2023 outbreak wave in Asia. The third phase corresponded to the emergence of clade Ib, detected from late 2024 to early 2025 based on publicly available sequences from Thailand deposited in GISAID/GenBank. All hMPXV genomes from Thailand deposited in public databases during this period belonged exclusively to clade Ib. The 16 samples in this study predated this emergence, as all were collected between 2023 and 2024 during the C.1‐dominated second phase.

### 3.3 Phylogenetic Structure of the Local C.1 Outbreak in Thailand

To resolve the fine‐scale phylogenetic structure within the C.1‐dominated second phase, subsequent analyses focused on sub‐lineage classification of Thai C.1 genomes. All 16 genomes generated in this study belonged to lineage C.1, with one genome further classified within the C.1.1 sub‐lineage. For comparative sub‐lineage analysis, these sequences were analyzed with additional publicly available Thai C.1 genomes (21 sequences) and global C.1 reference sequences (42 sequences) to contextualize the Thai outbreak within the broader C.1 phylogeny (SupportingData 3).

**Figure 2:**
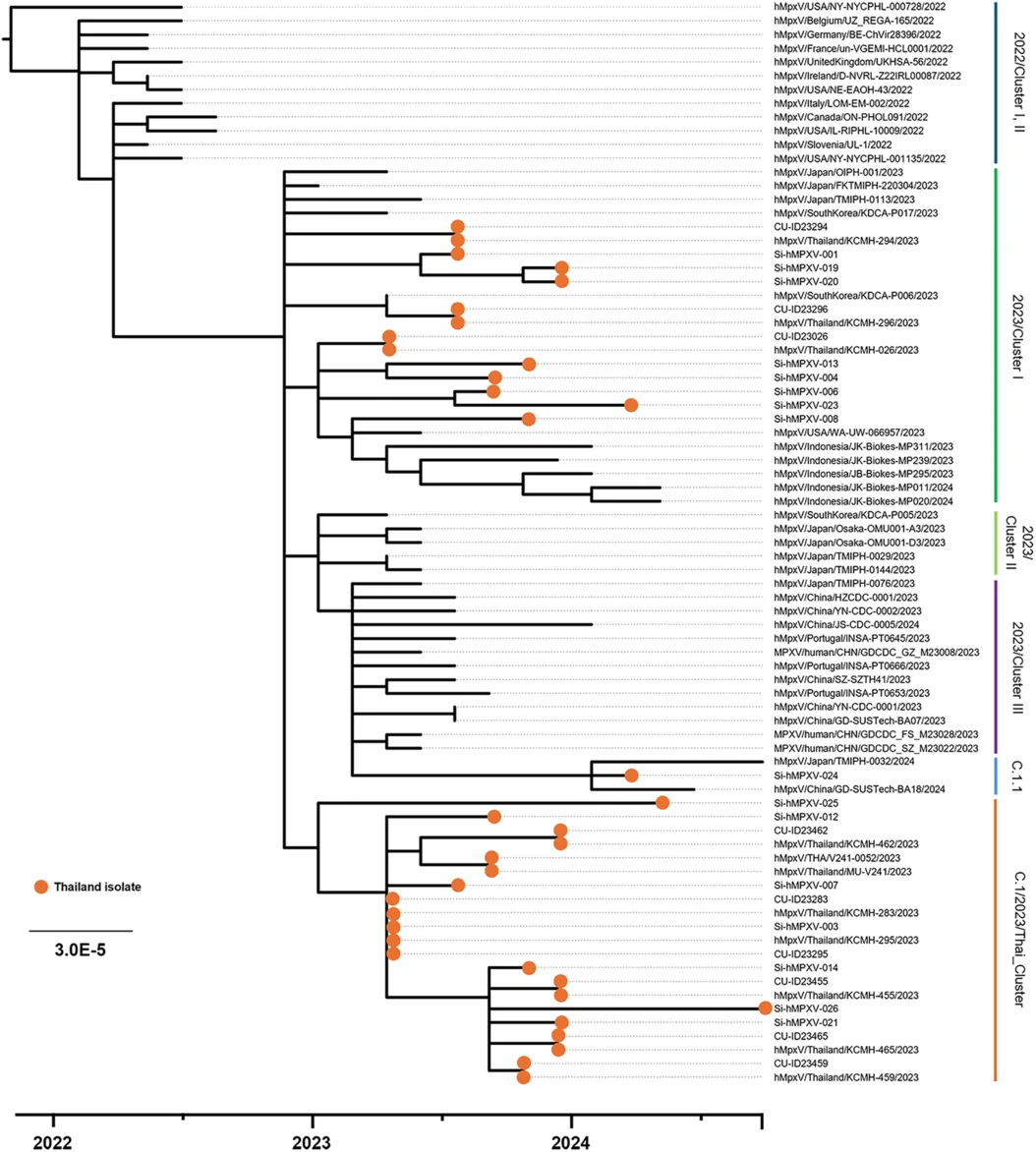
Time‐scaled phylogenetic analysis of hMPXV lineage C.1 including Thai isolates. Time‐scaled maximum‐likelihood phylogenetic tree of monkeypox virus (MPXV) lineage C.1 inferred from whole‐genome sequences and visualized using TreeTime. The tree includes 16 Thai hMPXV genomes generated in this study (Si‐hMPXV), additional Thai C.1 genomes from GISAID and GenBank, and representative global C.1 reference sequences. Thai isolates are highlighted as orange circles. The x‐axis represents calendar time.

To distinguish potential importation events from sustained local transmission, we applied the sub‐lineage clustering framework proposed by Yu et al. [24], which stratifies C.1 viruses into multiple temporal–phylogenetic clusters: C.1/2022/Cluster I, C.1/2022/Cluster II, C.1/2023/Cluster I, C.1/2023/Cluster II, and C.1/2023/Cluster III. Thai C.1 genomes were mapped onto this reference clustering scheme to facilitate comparative interpretation (Figure 2).

Phylogenetic reconstruction demonstrated that Thai C.1 genomes collected in 2023 and 2024 (*N* = 37, including 16 samples from this study) segregated into two distinct clusters. The first cluster grouped within C.1/2023/Cluster I and comprised 14 Thai genomes (8 from this study, 6 from public databases), which showed close phylogenetic relatedness to hMPXV strains reported from other Asian countries, including Japan and South Korea. Although the phylogenetic topology suggested potential regional introduction events, individual international travel histories, documented exposure events, and epidemiological links were unavailable for these patients. Therefore, the geographic source and direction of transmission could not be determined from genomic data alone.

In contrast, the second cluster comprised exclusively Thai hMPXV genomes sampled from mid‐2023 through 2024 and formed a phylogenetically distinct Thai‐specific cluster (C.1/Thai/Cluster). This group did not correspond to any of previously defined clusters proposed by Yu et al. and was clearly separated from globally described C.1/2023/Cluster II and III lineages. During mid‐2023, while hMPXV genomes from other countries diversified into C.1/2023/Cluster II and C.1/2023/Cluster III, Thai genomes instead gave rise to a separate C.1/Thai/Cluster. This cluster remained clearly distinct from other sampled Asian C.1 genomes, including those from China, Japan, South Korea, and Indonesia, and may reflect sustained local transmission within Thailand during this period. A single Thai genome (Si‐hMPXV‐024) clustered within the C.1.1 sub‐lineage, which has been predominantly reported in Europe and parts of Asia during 2023. The phylogenetic placement of this isolate suggests an independent introduction of C.1.1 into Thailand; however, limited sampling density and incomplete epidemiological metadata preclude further resolution of its transmission origin. Together, these findings indicate that the mpox outbreak in Thailand during 2023–2024 involved the circulation of multiple C.1 lineages, alongside the establishment of a Thailand‐specific C.1 transmission cluster (21 genomes, sampled between June 2023 and October 2024), consistent with sustained local transmission.

### 3.4 Mutation Spectrum and APOBEC3 Mutation

The 16 hMPXV sequences from this study belonged to the lineage C.1 and segregated into three phylogenetic groupings: C.1/2023/Cluster I, a C.1/Thai/Cluster, and C.1.1 lineage. To characterize the mutational processes shaping viral diversification and to identify mutations underlying the observed cluster structure and evolutionary patterns from isolated in this study, we analyzed nucleotide substitutions relative to the closely related Clade IIb reference strain MPXV_USA_2022_MA001 (GenBank accession ON563414.3), which is phylogenetically proximal to the C.1 lineage.

**Figure 3:**
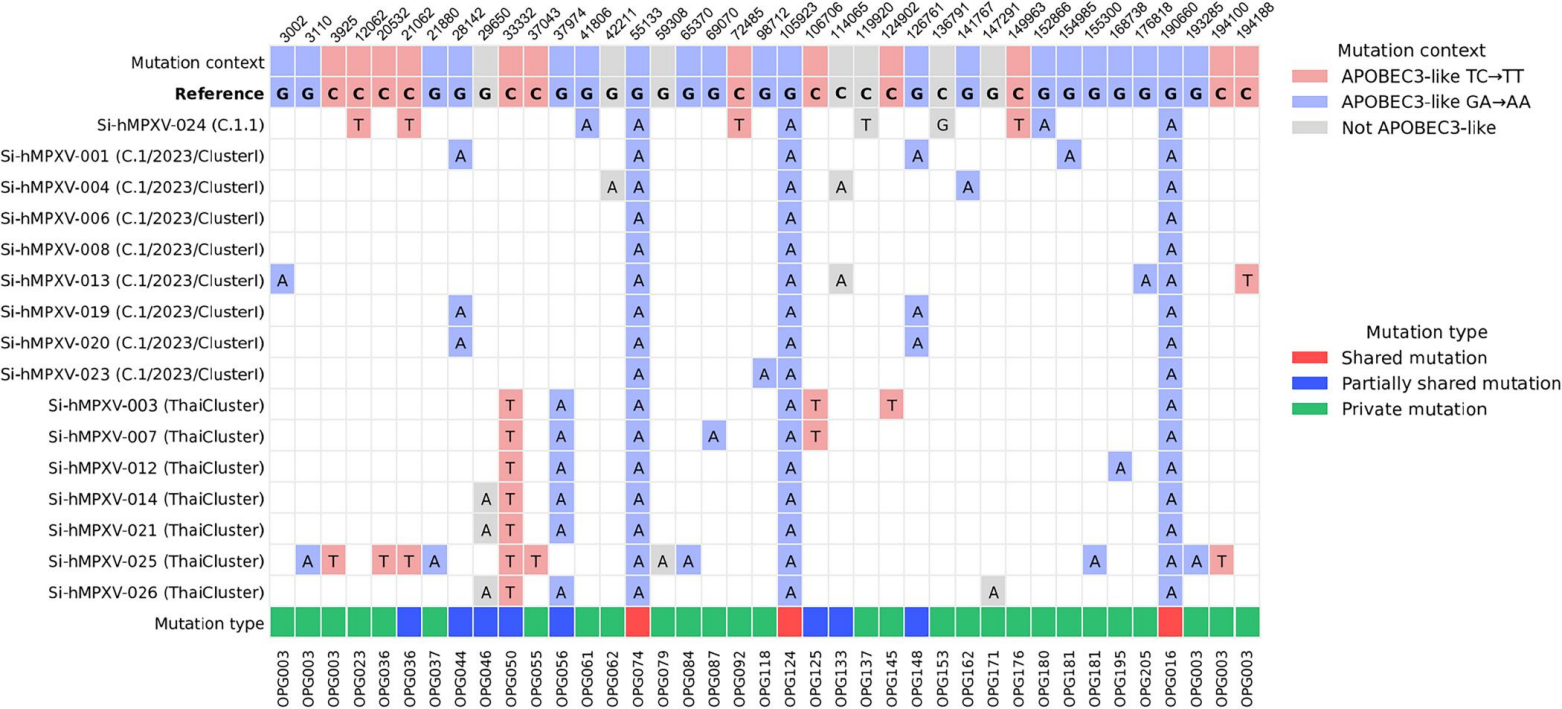
Mutation profile of selected coding‐region mutations across the 16 hMPXV isolates in this study. A total of 39 coding‐region SNPs were visualized, selected from 77 identified variants based on biological and phylogenetic relevance, including nonsynonymous changes, recurrence across multiple genomes, phylogenetics cluster‐informative variants, and substitutions consistent with APOBEC3‐associated signatures. Mutation positions are shown relative to the Clade IIb B.1 reference genome MPXV_USA_2022_MA001 (GenBank accession ON563414.3), in NC_063383.1 genomic coordinates. Colors represent nucleotide substitution types and mutational contexts, while annotation bars indicate mutation‐sharing patterns across genomes. Shared mutations denote variants present in all 16 isolates, partially shared mutations indicate variants detected in more than one but not all isolates, and private mutations represent variants unique to a single isolate.

Mutation profiling encompassed both synonymous and nonsynonymous substitutions, with particular emphasis on APOBEC3‐associated mutational signatures, specifically GA → AA and TC → TT transitions. Across the 16 isolates, a total of 77 distinct SNPs were identified relative to the IIb B.1 reference genome (ON563414.3), reported using NC_063383.1 genomic coordinates. For clarity and interpretability, 39 coding‐region SNPs were selected for visualization (Figure 3). Variants were considered relevant for visualization if they were non‐synonymous or predicted loss‐of‐function mutations. Intergenic variants and synonymous substitutions were excluded from the main figure. Mutation‐sharing patterns across genomes (shared, partially shared, or private) and consistency with APOBEC3‐associated mutational patterns (GA → AA or TC → TT) are indicated in the figure as annotations. Nonetheless, the complete set of 77 identified mutations is provided in the Supporting data 4. Of the 77 identified SNPs, 65 (84.4%) were consistent with APOBEC3‐associated mutational signatures (25 TC → TT and 40 GA → AA transitions), indicating that APOBEC3‐driven deamination represents the dominant mutational process shaping genomic diversification within the C.1 lineage. Notably, the majority of these non‐synonymous substitutions, including all three lineage‐defining shared mutations (OPG074, OPG124, OPG016), were consistent with APOBEC3‐associated mutational signatures, suggesting that host‐mediated deamination, rather than positive selection, may account for much of the amino acid‐altering variation observed across functional gene categories in this dataset.

Based on the distribution of mutation shown in Figure 3, all 16 hMPXV isolates shared a common set of SNPs consistent with the C.1 lineage, indicating a shared evolutionary background across the dataset. Several shared mutations were detected in all genomes, supporting their role as lineage‐defining features of C.1. In addition, some mutations were shared by only a subset of isolates and contributed to finer phylogenetic resolution. In particular, nucleotide substitutions at positions 33,332 and 37,974, located in the OPG050 and OPG056 genes, respectively, were detected in most isolates belonging to the C.1/Thai/Cluster but were absent in Si‐hMPXV‐025. These partially shared mutations helped define the Thailand‐specific cluster and were consistent with the clustering pattern observed in Figure 2.

Evaluation of mutational signatures showed that APOBEC3‐associated substitutions (GA → AA and TC → TT) accounted for the majority of mutations across the C.1 lineage overall, without evidence of significant enrichment in the C.1/Thai/Cluster relative to other C.1‐related isolates. At the genome level, the mean number of APOBEC3‐consistent mutations was comparable between the C.1/Thai/Cluster (mean 12.6 per genome, 94.5% of total mutations) and C.1/2023/Cluster I (mean 11.6 per genome, 89.2% of total mutations), with no statistically significant difference (Mann‐Whitney U test, *p* = 0.68 for mutation count; *p* = 0.31 for proportion). Given the limited number of genomes per cluster (*n* = 7–8), this comparison should be interpreted as exploratory. These findings indicate that APOBEC3‐driven mutagenesis is a pervasive feature of C.1 lineage evolution overall, consistent with sustained human‐to‐human transmission, rather than a process specifically amplified within the Thailand‐specific transmission cluster.

At a more detailed phylogenetic level, private mutations provided additional resolution among subclusters. A nucleotide substitution at position 29,650 in the OPG046 gene was detected only in isolates Si‐hMPXV‐014, Si‐hMPXV‐021, and Si‐hMPXV‐026. This private mutation was consistent with the grouping of these isolates in the phylogenetic tree (Figure 2), supporting its value as a subcluster‐informative marker.

**Figure 4:**
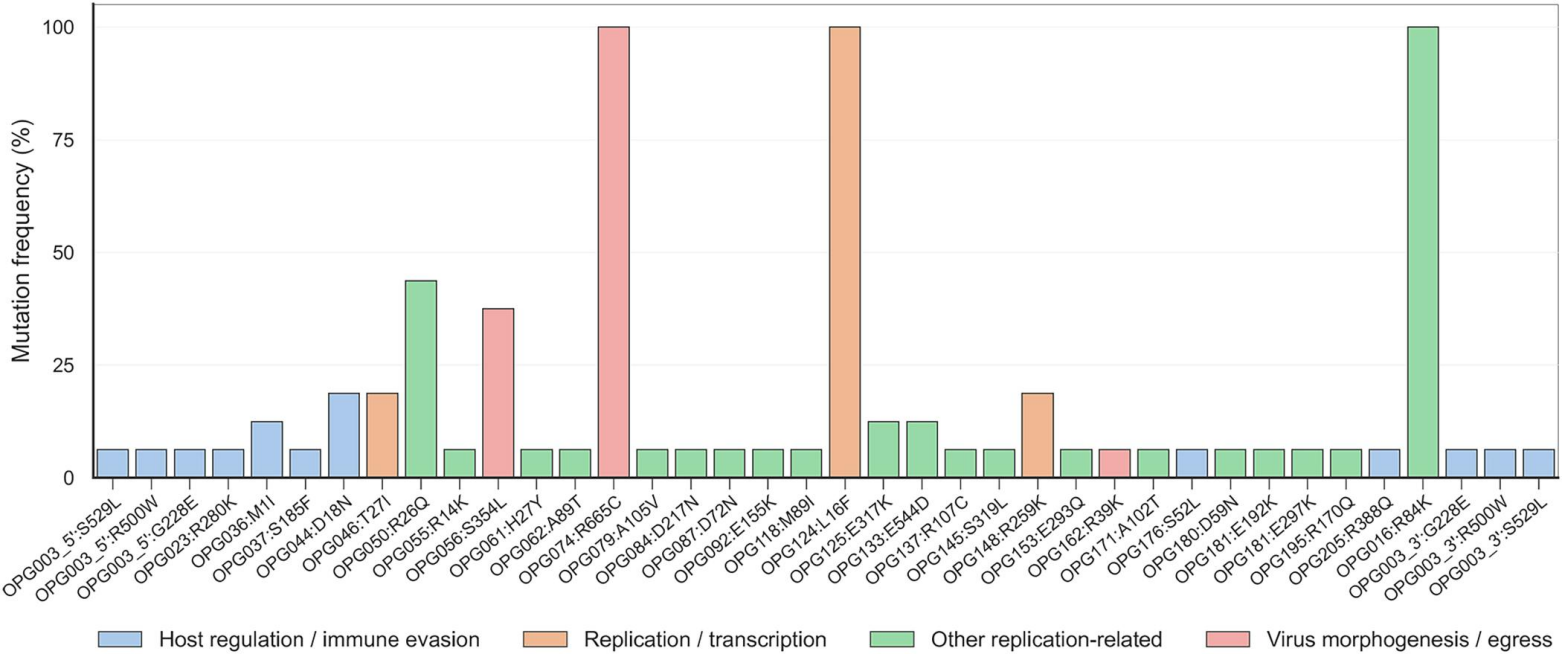
Mutation frequency of key non‐synonymous substitutions identified. The bar plot shows the frequency (%) of selected non‐synonymous mutations across the 16 isolates from this study. Mutations are displayed by gene (OPG) and amino acid substitution on the x‐axis, with frequencies calculated based on the total number of study isolates. Bars are color‐coded according to predicted functional categories, including host regulation/immune evasion, replication/transcription, other replication‐related functions, and virus morphogenesis/egress. Mutation positions are shown relative to the Clade IIb B.1 reference genome MPXV_USA_2022_MA001 (GenBank accession ON563414.3), using NC_063383.1 genomic coordinates.

To characterize the distribution of amino acid–altering variation among the studied hMPXV isolates, the frequency of non‐synonymous mutations identified in 16 genomes was examined across viral genes with different biological roles (Figure 4). Non‐synonymous mutations were observed in genes spanning multiple functional categories, including host regulation and immune evasion, viral replication and transcription, other replication‐related functions, and virus morphogenesis and egress. Shared non‐synonymous mutations were limited to a small number of genes and included OPG074 (R665C), OPG124 (L16F), and OPG016 (R84K), all of which were detected in all 16 isolates. These genes encode proteins associated with distinct functional categories, including virus morphogenesis and egress (OPG074), viral replication and transcription (OPG124), and other replication‐related functions (OPG016).

In contrast, partially shared non‐synonymous mutations were identified in several genes, including OPG050:R26Q, OPG056:S354L, OPG044:D18N, OPG046:T27I, and OPG148:R259K, which were present in a subset of the analyzed genomes. Private non‐synonymous mutations were detected in single isolates only and were distributed across a broad range of genes, including those involved in host interaction and immune modulation, such as OPG003 (G228E, R500W, S529L), OPG036:M1I, and OPG176:S52L, as well as genes associated with virus morphogenesis and egress, including OPG180:D55N and OPG205:R389Q.

Overall, the non‐synonymous mutation profile demonstrates that shared mutations were restricted to a small number of genes, whereas partially shared and private mutations were more widely distributed across functional categories, reflecting heterogeneity in the distribution of amino acid–altering substitutions among the analyzed hMPXV genomes.

### 3.5 Clinical Associations With Viral Mutations

Exploratory analyses were performed to assess potential associations between viral genetic variation and clinical outcomes. Individual mutation–outcome associations were initially evaluated using Fisher's exact test; however, none of the tested mutations remained statistically significant after correction for multiple comparisons. Accordingly, subsequent analyses focused on mutation groups and aggregate mutation burden, which are more appropriate for the limited sample size.

Severe disease was defined a priori based on clinically relevant manifestations, including hospitalization, disseminated cutaneous involvement affecting multiple non‐genital body sites, or the presence of perianal disease and/or proctitis. Patients not meeting these criteria were classified as having non‐severe disease. At the gene level, the presence of non‐synonymous mutations in selected viral genes, including OPG050:R26Q and OPG056:S354L, showed a trend toward higher frequency among patients classified as having severe disease compared with non‐severe cases. These mutations were found in C.1/Thai/Cluster. These associations were evaluated using Fisher's exact test with Haldane–Anscombe correction for sparse contingency tables, yielding odds ratios greater than one; however, the corresponding *p*‐values (*p* = 0.14 for OPG050:R26Q and *p* = 0.31 for OPG056:S354L) did not reach statistical significance after false discovery rate adjustment. No consistent associations were observed between mutations in other examined genes and hospitalization, disseminated rash, perianal involvement, or high viral load.

Analysis of overall mutation burden demonstrated that patients with severe disease exhibited a higher median number of selected non‐synonymous mutations (median = 8.0, IQR = 7.0–8.0) compared with those with non‐severe disease (median = 6.5, IQR = 6.0–7.0). These comparisons, assessed using the Mann–Whitney U test, indicated a directional difference but did not achieve statistical significance (*p* = 0.20). Similarly, neither APOBEC3‐associated mutation burden (*p* = 0.96) nor overall mutation burden (*p* = 0.61) was significantly associated with viral load, as estimated by PCR cycle threshold values. Consistent with this, when viral load was assessed using absolute quantification by ddPCR (log10 copies/mL), neither APOBEC3‐associated mutation burden (*p* = 0.51) nor overall mutation burden (*p* = 0.14) showed a statistically significant association, although a modest trend was observed for overall mutation burden. These findings indicate that while certain gene‐level mutation patterns and increased mutation burden exhibit trends suggestive of clinical relevance, no statistically robust associations were identified in this cohort. Given the limited sample size, these observations should be interpreted as hypothesis‐generating and warrant validation in larger, independent datasets.

## 4 Discussion

Historically, mpox was considered a rare and self‐limiting disease in humans; however, the disease has emerged as a global public health concern due to its increased prevalence outside endemic regions [25]. The 2022–2023 outbreak marked a significant shift in the epidemiology of the disease, with widespread human‐to‐human transmission reported, particularly among MSM populations [26]. The outbreak revealed new patterns of transmission, with intimate physical and sexual contact playing a key role in the spread of the virus [27]. In Thailand, mpox has been reported, but there is a lack of comprehensive clinical and genetic data to characterize the outbreak fully [12]. This gap in knowledge limits understanding of transmission dynamics, clinical severity, and viral evolution in the region. This study provides a comprehensive analysis of clinical and genetic characteristics of confirmed mpox cases diagnosed between 2023 and 2024, which was dominated by clade IIb lineage C.1. Phylogenetic analyses indicated a transmission pattern involving regional introductions followed by sustained local transmission. Clinically, cases occurred predominantly among MSM and were characterized by frequent HIV coinfection and genital or painful lesions. Despite the observed viral genetic diversity, exploratory analyses identified no statistically robust associations between specific mutations, APOBEC3‐associated substitutions, mutation burden, and clinical severity.

The demographic data revealed that all confirmed mpox cases in this study occurred in males, with a mean age of 39.29 years, and all identified as MSM. This aligns with global reports indicating that mpox disproportionately affects MSM in the current outbreak [26]. These findings emphasize the importance of targeted education and public health campaigns aimed at MSM communities. Furthermore, our finding of a high prevalence of HIV co‐infection (81.25%) is consistent with reports from other regions, including a multi‐country study that reported HIV prevalence among mpox patients as ranging from 40% to 60% [28]. The immune suppression observed in some patients may have contributed to the clinical presentation and severity of mpox, as suggested by previous studies [29]. However, in our study we found that 81.25% of HIV infected patients demonstrated HIV viral load less than 40 copies/mL, indicating that they were virally suppressed individuals. The high rate of HIV coinfection in our study may attribute to an increase in high risk behavior among these patients. The clinical presentation of mpox in this study was consistent with previously reported cases, with umbilicated pustules and fever being the most common symptoms, followed by ulcers and lymphadenopathy [30]. Notably, the prevalence of painful lesions (75%) and their localization to the genitalia (68.75%) are comparable to findings reported in European and North American study [31]. These findings highlight the need for clinicians to maintain a high index of suspicion for mpox in patients presenting with genital and perianal lesions, particularly in MSM populations. However, the high prevalence of concurrent STIs, such as syphilis (56.25%) and hepatitis B (25%), observed in our study, exceeds rates documented in some prior studies, underscoring the syndemic nature of mpox in this population and the need for integrated STI screening and management [32, 33]. Hospitalization rates in this study (25%) are within the range reported globally, where severe cases requiring inpatient care were observed in approximately 10%–30% of patients, depending on immune status and presence of co‐infections [29]. Using tecovirimat for hospitalized patients aligns with current treatment recommendations and has been shown to reduce disease severity in other studies [34]. In our study, mpox infection showed a favorable outcome with 93.75% of the patients improved and 0% mortality after treatment which aligns with previous study that reported mpox as self‐limiting with mortality at about 1‐3% [35].

The phylogenetic reconstruction provides a coherent overview of the temporal progression of hMPXV transmission in Thailand and places the study isolates within the broader regional and global context. The early phase of the Thai outbreak in mid‐2022 was characterized by the presence of clade IIb lineages A and B, with Thai sequences interspersed among global references. This pattern is consistent with multiple independent introductions and limited sustained local transmission, as has been reported during the initial expansion of mpox outside endemic regions [36, 37].

In contrast, the second phase, spanning 2023 to 2024, was dominated by lineage C.1 and its descendant C.1.1, during which all genomes generated in this study were collected. The clustering of Thai C.1 genomes with contemporaneous sequences from other Asian countries suggests regional circulation of this lineage rather than isolated, country‐specific evolution. Similar regional clustering of C.1 has been described in Asia during the post‐2022 outbreak period, reflecting ongoing transmission within this lineage [24, 38, 39]. Although the samples analyzed in this study predated the emergence of clade Ib, this apparent lineage turnover highlights the dynamic nature of mpox transmission in Thailand. In this context, the availability of genomic data following the emergence of clade Ib appears to be largely limited to this lineage, potentially reflecting a surveillance focus on newly emerging variants. Such an approach, valuable for early detection of emerging lineages, may limit the ability to resolve the ongoing circulation and diversification of established lineages within the country. These observations highlight the need for sustained, broad genomic surveillance to fully capture the evolving diversity of hMPXV over time [40].

Fine‐scale phylogenetic analysis of lineage C.1 revealed a heterogeneous transmission structure underlying the mpox outbreak in Thailand during 2023 to 2024. By applying a previously described sub‐lineage clustering framework for C.1 virus [24], Thai‐hMPXV genomes could be resolved into distinct phylogenetic groupings that are informative for distinguishing between potential importation events and subsequent local transmission. hMPXV from Thailand collected in 2023 segregated into two major phylogenetic clusters. One cluster, grouped within C.1/2023/Cluster I, showed close relatedness to contemporaneous C.1 strain reported from other Asian countries, suggesting regional connectivity in viral circulation. This finding is similar to the previous finding that found the most lineage IIb‐C of hMPXV in Thailand [36].

In contrast, a second cluster consisted exclusively of Thai hMPXV genomes collected from mid‐2023 to 2024 and was phylogenetically distinct from C.1 lineage circulating in other regions. This pattern is consistent with sustained local transmission following an initial introduction. A single isolate belonging to the C.1.1 sub‐lineage suggests an additional independent introduction. However, limited sampling and incomplete epidemiological data prevent further resolution. Overall, these findings suggest that the 2023–2024 mpox outbreak in Thailand involved both regional introductions and subsequent local transmission, resulting in the establishment of C.1/Thai/Cluster. However, these findings should be interpreted cautiously because phylogenetic analysis does not, by itself, establish the geographic origin or direction of transmission. Detailed travel histories, exposure events, and epidemiological links were not consistently available in the retrospective anonymized dataset; consequently, potential introduction events could not be confirmed. In addition, temporal structure was inferred using TreeTime, a maximum‐likelihood approach that estimates divergence times on a fixed topology without the posterior uncertainty quantification provided by full Bayesian methods (e.g., BEAST2). Future studies employing Bayesian phylodynamic inference with explicit coalescent models would allow more rigorous estimation of the timing and directionality of transmission events within the C.1 outbreak.

Exploratory analyses did not identify statistically significant associations between individual viral mutations and clinical severity after correction for multiple comparisons. Although selected non‐synonymous mutations within the C.1/Thai/Cluster, including OPG050:R26Q and OPG056:S354L, were more frequently observed among patients with severe disease, these associations did not reach statistical significance and should be interpreted cautiously.

Similarly, patients with severe disease exhibited a higher median non‐synonymous mutation burden than non‐severe cases, but this difference was not statistically significant. No consistent associations were observed between mutation burden, APOBEC3‐associated substitutions, or viral load, whether estimated by PCR cycle threshold values or by absolute quantification using ddPCR. These findings may reflect the limited statistical power available in a cohort of this size, as well as the likelihood that clinical severity in mpox is multifactorial, shaped by host immune status and other host‐related factors rather than viral genetic variation alone. This interpretation is consistent with previous mpox studies suggesting that host‐related factors, rather than viral genetic variation, are more likely to influence clinical severity [6]. Given the limited sample size and exploratory nature of the analyses, the observed trends are best regarded as hypothesis‐generating and warrant validation in larger studies integrating viral genomics with detailed clinical and host‐related data.

In summary, this study provides the whole‐genome‐based characterization of hMPXV circulating in Thailand and demonstrates that the 2023‐2024 outbreak was dominated by lineage C.1, with evidence supporting both regional introductions and sustained local transmission. Fine‐scale phylogenetic analysis identified a Thailand‐specific C.1 cluster, indicating local amplification following an initial introduction, while mutation profiling showed that viral diversification was predominantly shaped by APOBEC3‐associated mutagenesis, consistent with sustained human‐to‐human transmission, rather than cluster‐specific adaptive processes. Although exploratory analyses suggested trends between viral genetic features and clinical severity, no statistically robust genotype‐pheotype associations were identified, underscoring the likely contribution of host and epidemiological factors to disease outcome. Together, these findings highlight the value of integrating genomic and clinical data for understanding mpox transmission dynamics and emphasize the importance of sustained, comprehensive genomic surveillance to monitor both emerging and established hMPXV lineages in Thailand.

## Author Contributions

Conceptualization: Kamol Suwannakarn, Navin Horthongkham. Study design and supervision: Navin Horthongkham. Clinical investigation and sample collection: Charussri Leeyaphan, Pattriya Jirawattanadon, Pravarit Athiprayoon. Laboratory investigation: Kamol Suwannakarn, Natsada Saengdao, Piyarat Taiphakphaibun, Chutima Deetippasert, Warinya Nuwong, Niracha Athipanyasilp, Archiraya Pattama. Data curation and formal analysis: Kamol Suwannakarn, Chutikarn Chaimayo, Atsadang Boonmee, Navin Horthongkham. Writing – original draft: Kamol Suwannakarn. Writing – review and editing: Kamol Suwannakarn, Chutikarn Chaimayo, Atsadang Boonmee, Charussri Leeyaphan, Pattriya Jirawattanadon, Navin Horthongkham. All authors reviewed and approved the final manuscript.

## Ethics Statement

The study protocol was approved by the Siriraj Institutional Review Board, Mahidol University (COA 941/2024), and was conducted in accordance with the principles of the Declaration of Helsinki. The requirement for informed consent was waived by the Institutional Review Board because all specimens and associated clinical data were anonymized.

## Conflicts of Interest

The authors declare no competing interests. Sequencing reagents used in this study were provided by MGI Tech Co. Ltd. The company had no role in the study design, data collection, analysis, interpretation of the results, or the decision to submit the manuscript for publication.

## Declaration of Generative AI and AI‐Assisted Technologies in the Writing Process

During the preparation of this work, the authors used ChatGPT (model GPT‐5.2; OpenAI) to assist with English language editing. Following the use of this tool, the authors reviewed and edited the content as necessary and take full responsibility for the integrity and accuracy of the final manuscript.

## Supporting information


**Figure S1:** Time to cure in all patients

Supporting File 1

Supporting File 2

Supporting File 3

Supporting File 4

## Data Availability

The genome sequences generated in this study have been deposited in GenBank under accession numbers PZ055577‐PZ055592. Additional data supporting the findings of this study are available from the corresponding author upon reasonable request.
