# Supplementary material for: Genomic Epidemiology and Clinical Characteristics of Mpox Lineage C.1 Outbreak in Thailand, 2023–2024
Source: J Med Virol. 2026 Sep 8;98(9):e71142. doi: 10.1002/jmv.71142 (PMC13552223; doi:10.1002/jmv.71142)
Supplement: Supplementary file 1 [file JMV-98-e71142-s003.docx]

**Supplementary Figure 1.** Time to cure in all patients
